# Has the COVID-19 pandemic altered social inequalities in child development? A time trend analysis of developmental delays in preschool children by parental education

**DOI:** 10.1186/s13690-026-02111-x

**Published:** 2026-10-15

**Authors:** Timo-Kolja Pförtner, Thomas Hennemann, Monika Gube, Frank J. Elgar

**Affiliations:** 1 Child and Youth Welfare and social-emotional development advancement, Department of Rehabilitation and Special Education, Faculty of Human Science, University of Cologne, Cologne, Germany; 2 Health Authority of the City and Area of Aachen, Aachen, Germany; 3 Institute for Health and Social Policy, McGill University, Montreal, QC Canada

**Keywords:** COVID-19 pandemic, child development, parental education, socioeconomic status, time trend

## Abstract

**Background:**

Child development may have deteriorated during the COVID-19 pandemic, with children from vulnerable backgrounds potentially facing the greatest risks. This study examined time trends in developmental delays among preschool children and whether inequalities in these delays by parental education changed with the onset of the pandemic.

**Methods:**

Analyses used mandatory School Entry Examinations (SEE) in the Aachen region, Germany, covering ten cohorts (2015–2024) and restricted to children identified as being at risk for developmental delays (*n* = 15,299). The outcome was the number of delayed domains among six assessed. A segmented (interrupted time series) Poisson regression estimated the pre-pandemic trend, the level shift at the break between the 2020 and 2021 cohorts, and the subsequent change in trend, with inequalities by parental education expressed on both the relative and absolute scale. Multiple imputation and inverse probability weighting were applied.

**Results:**

Children of less-educated parents showed significantly more delays in every cohort. Delays increased significantly before the pandemic at a comparable rate in all educational groups. Delays rose abruptly in the 2021 cohort in all groups and declined thereafter, remaining elevated. Between the two periods, relative inequality narrowed significantly because delays increased proportionally more among children with moderate and high parental education, whereas absolute inequality was unchanged.

**Conclusion:**

Children from families with lower educational attainment continued to experience a consistently higher burden of developmental delays. The first cohort assessed after the onset of the pandemic showed an abrupt, population-wide increase, which reduced inequality on a relative scale while leaving the absolute gap between educational groups unchanged. Public health strategies must address both persistent structural disparities and the broad vulnerabilities revealed by global crises.

**Supplementary Information:**

The online version contains supplementary material available at https://doi.org/10.1186/s13690-026-02111-x.

**Table:** 

Text box 1. Contributions to the literature		
• Children of less-educated parents showed persistently more developmental delays across all ten cohorts.		
• Delays increased in every educational group and rose abruptly in the first cohort examined after the onset of the pandemic.		
• Relative inequality reduced significantly, whereas the absolute gap between educational groups remained unchanged.		
• The reduction follows from unequal baseline levels rather than from a smaller impact on disadvantaged children.		
• These insights can inform public health strategies and school-entry interventions to address both long-standing structural inequalities and the specific developmental setbacks emerging from large-scale societal disruptions.		

## Introduction

The COVID-19 pandemic, first reported in late 2019 and declared a pandemic by the World Health Organization in March 2020, disrupted societies worldwide. In response, countries such as Germany implemented public health measures including the closure of educational facilities, social distancing, and mask mandates [1]. These measures may have negatively impacted the development of preschool children [2–4]. Crucial factors for healthy development, such as opportunities for physical activity, preschool support, stress regulation, and social interaction with peers and professionals, were severely restricted during this period [5–9]. Studies have indicated that family stress, mental health problems, family conflicts, and media consumption increased during the pandemic, leading to heightened emotional distress and anxiety and a decline in social skills among preschool children [7, 10–16].

The profound disruptions caused by the pandemic may have affected early childhood development differently depending on parental education. Operationalized as the highest level of education attained by either parent [17], parental education is associated with human (e.g., parental skills), cultural (e.g., preferences for high culture), and social capital (e.g., network connections) that jointly shape the environment for child development [18, 19]. Parental education is also associated with genetic and socioeconomic influences on early child development, including household income and family background [19]. It is, however, only one dimension of socioeconomic status and is not interchangeable with income or occupational status, which may operate through partly different mechanisms [18]. Existing research consistently demonstrates inequalities in child development to the disadvantage of children with lower parental education [20–22].

Theoretically, the pandemic could have moved inequalities in three directions. They would widen if low parental education became a greater constraint through fewer resources to adapt to public health measures and greater intrafamilial stress from illness, job loss, financial strain, or overcrowded housing [3, 8, 23, 24]. Furthermore, inequalities would also widen because closures removed an institutional environment that ordinarily compensates for unequal home resources [25], with pandemic-related learning losses largest among children from less-educated households [26–28]. Inequalities would narrow if better-educated families were more affected by the pandemic, as, for example, the closures removed institutional and extracurricular provision that these families use more intensively [29, 30], and as emergency care was reserved for groups that included socially disadvantaged children [10]. They would remain unchanged if the disruption operated through a channel common to all families, such as the general restriction of contact with peers and of access to public play and leisure facilities, or if the mechanisms working in opposite directions offset one another. Which of these is observed also depends on the measure, since relative and absolute measures of inequality can move in opposite directions when baseline levels differ [31, 32]. Evidence regarding the impact of the COVID-19 pandemic remains mixed. A study from Uruguay reported significantly greater losses in motor and cognitive development among socioeconomically disadvantaged children aged 4–6 years [33]. In contrast, data from school entry surveys in Düsseldorf, Germany, revealed that language problems increased more in socioeconomically advantaged neighborhoods, while coordination problems rose equally across all groups [29]. Furthermore, reports from parents and pediatricians in Germany indicated that negative effects on the social and emotional development of infants were stronger among families living in poverty [34]. These studies are difficult to compare, as they differ in the children’s age, in the domains assessed, in whether development was reported by parents or assessed directly, and in the indicator of social position. They also refer to different phases of the pandemic and to countries that differed in their containment measures and public communication [1, 35]. Their findings should therefore not be interpreted as evidence of a single pandemic effect on child development.

Despite these findings, current research lacks comprehensive analyses of time trends in social inequalities in developmental delays, specifically concerning the COVID-19 pandemic, with existing studies covering few cohorts and reporting inequality on a single scale. A design that applies the same standardized assessment to consecutive cohorts before and after the pandemic onset, and that reports inequality on both scales, can therefore address a question the existing evidence leaves open. This study examines whether social inequalities in preschool child development changed with the onset of the COVID-19 pandemic, using data from the School Entry Examination (SEE) in the Aachen region, Germany. The Aachen region, located in North Rhine-Westphalia, is one of Germany’s economically disadvantaged regions, where poverty has steadily increased over time [36]. In this region, pandemic containment measures followed federal and state legislation [37, 38]. Comprehensive restrictions were introduced at the onset of the pandemic and during the second wave in winter 2020/2021, including the closure of schools and childcare facilities. However, emergency care remained accessible for children with diagnosed disabilities, children from families receiving social welfare, children of single parents, and those whose parents worked in systemically relevant professions [10]. Following the introduction of comprehensive testing and vaccination options, closures of entertainment facilities, schools, and childcare facilities were eased in spring 2021. All legal regulations still in place to contain the COVID-19 pandemic were successively relaxed and expired completely in spring 2023.

The following analyses focus on children identified by kindergartens or schools as being at risk for developmental delays, because the Aachen public health department restricted the examination of the 2021 school entry cohort to this group in response to pandemic-related regulations. The aims of this study were to investigate (1) how developmental delays among at-risk children aged 4 to 7 years changed between 2015 and 2024; (2) whether these time trends differed by parental education; and (3) whether the onset of the pandemic marked a discontinuity in educational inequalities in developmental delays, distinguishing an abrupt shift in level from a change in the subsequent trend, and assessing inequality on both the relative and the absolute scale.

## Methods

### Study population

This study utilized data from the mandatory SEE conducted between 2015 and 2024 in the Aachen region of Germany. In Germany, the SEE is a compulsory assessment designed to evaluate a child’s physical, mental, and health readiness for school. It serves to identify disabilities or developmental delays, provide guidance on diagnostic and therapeutic measures, and recommend appropriate school placements or adjustments.

The SEE is conducted by the Health Authority of the City and Area of Aachen, typically two to twelve months before school entry. The annual school enrollment cohort in Aachen comprises approximately 4,800 children, primarily aged 5–6 years. The examination includes the collection of demographic data, a review of vaccination status and medical history, and assessments of vision, hearing, and developmental status. A physical examination and a parental counseling interview complete the assessment. The SEE is conducted between August of the year preceding school entry and July of the school entry year. Children reported by kindergarten or school staff as showing developmental risks are scheduled first, enabling the health authority to forward the SEE report to the receiving school before the deadline for special educational needs procedure (AOSF) applications on January 20. The identification of these potentially at-risk children is not reliant on a specifically developed standardized test screening but rather on the observation and experience of kindergarten and school staff. Detailed information on the SEE data collection protocol is available in the literature [39]. All the children’s data were anonymized before the analyses. The underlying data for this study are maintained by the Health Authority of the City and Area of Aachen and can be accessed upon reasonable request for research purposes, provided that ethical and legal requirements are met.

The analyses focused exclusively on children reported at risk of developmental delays to ensure comparability across the various years of SEE. In the school entry year 2021, the Health Authority of the City and Area of Aachen assessed only those children who had been reported by kindergartens or schools before SEE as potentially being at risk. In all other years, when the entire cohort was examined, at-risk children were identified by an examination date on or before January 31, shortly after the AOSF deadline and allowing for rescheduled appointments. Since the exact cut-off is to some extent arbitrary, sensitivity analyses varied it from December 31 to February 28 and additionally used a fixed proportion of each cohort instead of a fixed date. Because no examinations took place between March and August 2020, the 2021 cohort started later, and around 670 of its children were examined after January 31. This deviation is accounted for by a dummy variable in all models.

The percentage of children at potentially greater risk of developmental delays was approximately 32% between 2015 and 2024 (*n* = 15,299) (Table 1). Missing values were addressed by multiple imputation by chained equations (MICE), carried out on the full school entry cohort (*N* = 48,121) [40]. The imputation model included all variables entering the analyses, conditional on the fully observed predictors school entry year interacted with at-risk status, sex, and an indicator for children of the 2021 cohort examined after the regular cut-off date. Item-level missingness ranged from 0.4% to 14.9%. Thirty imputed datasets were generated, following the recommendation that the number of imputations should at least approximate the largest fraction of missing information expressed as a percentage [41]. Estimates were pooled using Rubin’s rules with the corresponding degrees-of-freedom adjustment [42]. Imputation left the distribution of covariates largely unchanged but raised the mean number of delays from 1.44 to 1.56, indicating that children with incomplete examinations had more delays than those with complete records (see supplementary Table S1).

**Table 1:** Characteristics of the analytic sample (School entry survey 2015 to 2024, Aachen region, Germany)

School entry year	Overall (*n* = 15,299)	2015 (*n* = 1,397)	2016 (*n* = 1,289)	2017 (*n* = 1,269)	2018 (*n* = 1,554)	2019 (*n* = 1,520)	2020 (*n*=,1 593)	2021 (*n* = 1,421)	2022 (*n* = 1,604)	2023 (*n* = 1,788)	2024 (*n* = 1,864)
Total *n* (%)											
Valid	13,015 (85.1)	1,107 (79.2)	1,069 (82.9)	1,038 (81.8)	1,330 (85.6)	1,144 (75.3)	1,367 (85.8)	1,259 (88.6)	1,456 (90.8)	1,601 (89.5)	1,644 (88.2)
Missing	2,284 (14.9)	290 (20.8)	220 (17.1)	231 (18.2)	224 (14.4)	376 (24.7)	226 (14.2)	162 (11.4)	148 (9.2)	187 (10.5)	220 (11.8)
Dependent variable											
Developmental delays M (SD)	1.44 (1.58)	1.19 (1.57)	1.24 (1.52)	1.34 (1.50)	1.26 (1.53)	1.41 (1.62)	1.40 (1.56)	1.66 (1.60)	1.62 (1.64)	1.52 (1.55)	1.63 (1.59)
Missing	2,284 (14.9)	290 (20.8)	220 (17.1)	231 (18.2)	224 (14.4)	376 (24.7)	226 (14.2)	162 (11.4)	148 (9.2)	187 (10.5)	220 (11.8)
Independent Variable											
Parental Education *n (%)*											
Low	4,643 (30.3)	386 (27.6)	398 (30.9)	404 (31.8)	484 (31.1)	484 (31.8)	432 (27.1)	475 (33.4)	493 (30.7)	526 (29.4)	561 (30.1)
Moderate	5,332 (34.9)	516 (36.9)	521 (40.4)	468 (36.9)	559 (36.0)	538 (35.4)	494 (31.0)	414 (29.1)	501 (31.2)	640 (35.8)	681 (36.5)
High	3,858 (25.2)	353 (25.3)	259 (20.1)	280 (22.1)	373 (24.0)	362 (23.8)	466 (29.3)	322 (22.7)	431 (26.9)	504 (28.2)	508 (27.3)
Missing	1,466 (9.6)	142 (10.2)	111 (8.6)	117 (9.2)	138 (8.9)	136 (8.9)	201 (12.6)	210 (14.8)	179 (11.2)	118 (6.6)	114 (6.1)
Covariates											
Sex *n* (%)											
Male	8,959 (58.6)	766 (54.8)	738 (57.3)	727 (57.3)	885 (56.9)	896 (58.9)	934 (58.6)	896 (63.1)	966 (60.2)	1 046 (58.5)	1 105 (59.3)
Female	6,340 (41.4)	631 (45.2)	551 (42.7)	542 (42.7)	669 (43.1)	624 (41.1)	659 (41.4)	525 (36.9)	638 (39.8)	742 (41.5)	759 (40.7)
Missing	-	-	-	-	-	-	-	-	-	-	-
Age in month M (SD)	68.0 (4.60)	68.3 (4.48)	67.8 (4.23)	68.4 (4.22)	67.9 (4.27)	68.4 (4.37)	67.8 (4.58)	69.7 (5.73)	67.4 (4.45)	67.4 (4.42)	67.3 (4.59)
Missing	10 (0.1)	2 (0.1)	-	3 (0.2)	2 (0.1)	1 (0.1)	-	-	1 (0.1)	1 (0.1)	-
Language in the first 4 life years *n* (%)											
German	7,895 (51.6)	802 (57.4)	710 (55.1)	685 (54.0)	829 (53.3)	797 (52.4)	832 (52.2)	662 (46.6)	793 (49.4)	897 (50.2)	888 (47.6)
Other	7,152 (46.7)	505 (36.1)	525 (40.7)	541 (42.6)	671 (43.2)	715 (47.0)	760 (47.7)	759 (53.4)	810 (50.5)	891 (49.8)	975 (52.3)
Missing	252 (1.6)	90 (6.4)	54 (4.2)	43 (3.4)	54 (3.5)	8 (0.5)	1 (0.1)	-	1 (0.1)	-	1 (0.1)
Family structure *n* (%)											
Two birth parents	10,749 (70.3)	945 (67.6)	901 (69.9)	860 (67.8)	1,110 (71.4)	1,044 (68.7)	1,175 (73.8)	950 (66.9)	1,145 (71.4)	1,267 (70.9)	1,352 (72.5)
Single parent	2,508 (16.4)	208 (14.9)	190 (14.7)	221 (17.4)	210 (13.5)	263 (17.3)	229 (14.4)	273 (19.2)	267 (16.6)	321 (18.0)	326 (17.5)
Birth parent with partner	640 (4.2)	58 (4.2)	67 (5.2)	50 (3.9)	75 (4.8)	76 (5.0)	69 (4.3)	64 (4.5)	63 (3.9)	61 (3.4)	57 (3.1)
Children’s home/Caregivers	261 (1.7)	38 (2.7)	21 (1.6)	25 (2.0)	23 (1.5)	20 (1.3)	31 (1.9)	35 (2.5)	26 (1.6)	26 (1.5)	16 (0.9)
Others	75 (0.5)	8 (0.6)	7 (0.5)	11 (0.9)	10 (0.6)	5 (0.3)	6 (0.4)	9 (0.6)	5 (0.3)	8 (0.4)	6 (0.3)
Missing	1,066 (7.0)	140 (10.0)	103 (8.0)	102 (8.0)	126 (8.1)	112 (7.4)	83 (5.2)	90 (6.3)	98 (6.1)	105 (5.9)	107 (5.7)
Nationality *n (%)*											
German	11,624 (76.0)	947 (67.8)	1,095 (84.9)	1,021 (80.5)	1,236 (79.5)	1,160 (76.3)	1,293 (81.2)	945 (66.5)	1,183 (73.8)	1,359 (76.0)	1,385 (74.3)
Other	2,323 (15.2)	98 (7.0)	134 (10.4)	148 (11.7)	211 (13.6)	265 (17.4)	236 (14.8)	246 (17.3)	285 (17.8)	320 (17.9)	380 (20.4)
Missing	1,352 (8.8)	352 (25.2)	60 (4.7)	100 (7.9)	107 (6.9)	95 (6.2)	64 (4.0)	230 (16.2)	136 (8.5)	109 (6.1)	99 (5.3)
Kindergarten attendance *n* (%)											
Yes	14,447 (94.4)	1,271 (91.0)	1,214 (94.2)	1,168 (92.0)	1,427 (91.8)	1,432 (94.2)	1,499 (94.1)	1,336 (94.0)	1,562 (97.4)	1,731 (96.8)	1,807 (96.9)
No	852 (5.6)	126 (9.0)	75 (5.8)	101 (8.0)	127 (8.2)	88 (5.8)	94 (5.9)	85 (6.0)	42 (2.6)	57 (3.2)	57 (3.1)
Missing	-	-	-	-	-	-	-	-	-	-	-

Observed data, unweighted and without imputation

### Delays in child development

Delays in child development are assessed across six domains considered central to coping with school requirements: physical coordination, selective attention, visual-motor skills, visual perception, knowledge of numbers and quantities, and speech and language. The school physician’s assessment draws on two sources (see supplementary Table S2). First, the “Sociopediatric developmental screening for SEEs” (SOPESS), a standardized and validated screening tool covering these domains [43–45]. Second, the physician’s qualitative assessment, incorporating previous findings or examinations. The final classification follows the “Bielefelder model” [46]. For each domain we coded a binary indicator taking the value one for a delayed ability, irrespective of whether treatment had already been initiated, or a diagnosed disability, and the value zero for an inconspicuous finding or a finding with few symptoms. Examinations that were not carried out or not carried out as intended were treated as missing and addressed by multiple imputation. The primary outcome was the number of developmental delays across these six domains (0–6), computed within each imputed dataset as the sum of the six domain indicators.

### Parental education

The measurement of parental education was based on information provided by the legal guardians on their schooling and vocational training, which we converted to an 8-point index: low (1–3), moderate (4–6), and high (7–8) (see supplementary Table S3) [47]. The highest level of parental education in a child’s household was considered in the analyses [19].

### Covariates

The covariates were the child’s age at examination (in months), sex, primary language spoken at home in the first four years of life (German or other), nationality (German or other), family structure (two birth parents, single birth parent, birth parent with partner, children’s home or foster care, others), and whether a kindergarten had ever been attended (yes or no). A further covariate identified children of the 2021 cohort who were examined after the usual January 31 cut-off.

### Statistical analyses

First, all models were weighted using stabilized inverse probability weights [48]. As the analytic sample was restricted to children identified as at risk, and this identification was likely associated with both parental education and child developmental status, selection bias may arise, particularly since the composition of this group varied across cohorts [49]. The weighting model was restricted to characteristics available for the entire cohort in all years. Selection was modeled by logistic regression on parental education interacted with school entry year, age, sex, nationality, family structure, and kindergarten attendance, with weights estimated within each imputed dataset and under the assumption that selection is not determined by unmeasured characteristics [50]. Weights ranged from 0.36 to 3.79 with a mean of 1.01, indicating no extreme selection probabilities. After weighting, characteristics included in the weighting model matched the full school entry cohort to within 0.5% points. Language spoken at home, which did not meet this criterion, moved from 11 to 7% points (see supplementary Table S1). The weights balance the composition of the at-risk sample across cohorts rather than extrapolating to children who were never reported. Estimates therefore continue to refer to children reported as at risk.

**Fig. 1:**
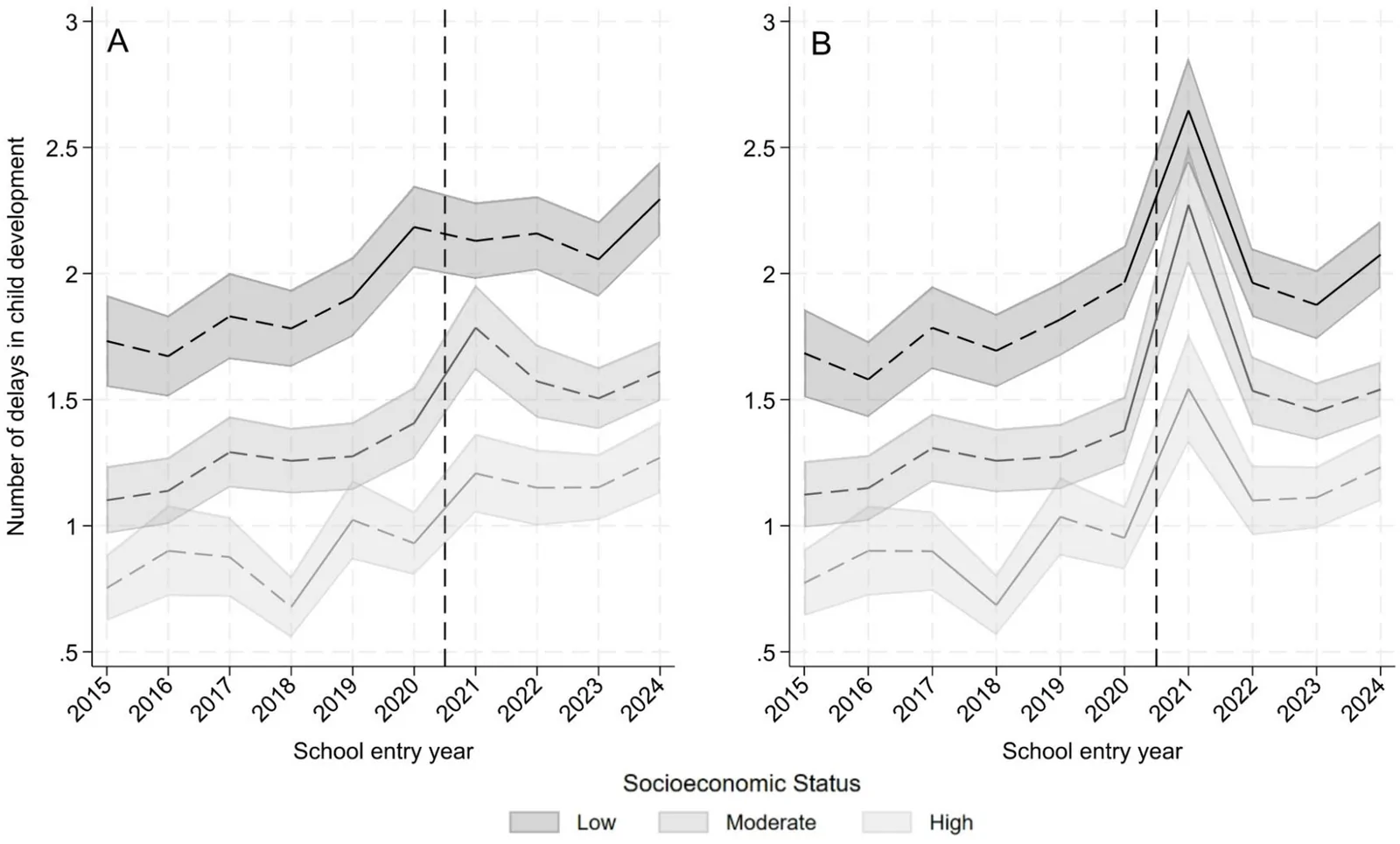
Numbers of delays in child development by parental education between 2015 and 2024, unadjusted (Panel **A**) and adjusted for sex, age in month, language in the first 4 life years, family structure, nationality, and kindergarten attendance (Panel **B**) (School entry survey 2015–2024, region of Aachen, Germany) (see Table S4 in the supplementary file). Notes: Values are model-based predictions of the expected number of delays, in Panel **B** standardized over the covariate distribution of the analytic sample. Estimates are weighted by inverse probability weights and pooled across 30 imputed datasets. Shaded areas denote 95% confidence intervals. The vertical dashed line marks the break between the last pre-pandemic cohort (2020) and the first pandemic cohort (2021). Solid line segments indicate a change from the preceding cohort at *p* ≤ .05 and dashed segments a change at *p* > .05, tested as contrasts of adjacent predictions

Second, the course of developmental delays by parental education was estimated from Poisson models with a separate parameter for each combination of cohort and education group, once without further adjustment and once with the full covariate set. From these models, adjusted predictions of the expected number of delays were computed, standardized over the covariate distribution of the analytic sample (Fig. 1) [51]. Changes between consecutive cohorts were tested within each education group as contrasts of these predictions. These comparisons were exploratory and were not corrected for multiplicity.

Third, the number of developmental delays was modeled using Poisson regression with robust standard errors in a segmented (interrupted time series) specification, adjusted for all covariates [52, 53]. The model included a linear trend across the pre-pandemic cohorts, a level shift beginning with the 2021 cohort, a change in slope from the 2021 cohort onward, and interactions between each of these terms and parental education. Individual time coefficients were reported descriptively, whereas inference on the time structure rested on joint hypothesis tests. These were used to assess whether the educational gradient was already changing before the pandemic, whether a shift occurred at its onset, and whether the gradient changed with the pandemic. All Wald tests were computed using multiple-imputation combining rules, with denominator degrees of freedom adjusted for the fraction of missing information.

Educational inequality was quantified on both the relative and absolute scales. Relative inequality was expressed as the ratio of expected counts of developmental delays between educational groups, and its change over time as the ratio of these ratios. Absolute inequality was expressed as the difference in expected counts and estimated as contrasts of adjusted predictions, standardized to the covariate distribution of the analytic sample. Both measures were evaluated at the child-weighted mean value of the cohort time variable within the pre-pandemic and pandemic periods, and changes between periods were tested as contrasts [54].

The six domains of developmental delays were analyzed separately using the same modeling specification to assess whether the findings for the summary score were consistent across developmental domains. For these analyses, p-values were additionally reported with Holm [55] and Benjamini–Hochberg [56] correction. Further sensitivity analyses used complete cases instead of multiple imputation, omitted the weights, applied five alternative definitions of the at-risk sample, and allowed for a nonlinear trajectory of the pandemic effect. The significance level was set at α = 0.05. Analyses were conducted in Stata SE 19.5 (StataCorp, College Station, TX, USA).

## Results

Table 1 presents descriptive information on the analytic sample. Of the 15,299 children reported at risk of developmental delays, 58.6% were boys, and children were on average 68.0 months old (SD 4.60) at examination. Most lived with two birth parents (70.3%) and had attended kindergarten (94.4%), while 46.7% had grown up with a language other than German in their first four years of life. Parental education was low for 30.3%, moderate for 34.9%, and high for 25.2% of the children, and missing for 9.6%. On average, children showed 1.44 developmental delays (SD 1.58) across the six domains, with this information missing for 14.9%. The proportion of boys rose from 54.8% in 2015 to 59.3% in 2024, the proportion of children with a language other than German from 36.1% to 52.3%, and the proportion living with a single parent from 14.9% to 17.5%, while kindergarten attendance increased from 91.0% to 96.9%. The distribution of parental education remained comparatively stable. In 2021, the mean age at examination was about two months higher than in the surrounding cohorts (69.7 months), and the mean number of delays was the highest observed over the entire period (1.66).

Figure 1 shows the expected number of developmental delays by parental education across school entry cohorts, weighted by stabilized inverse probability weights and presented without (Panel A) and with adjustment for all covariates (Panel B). In both panels, children from low education households consistently showed the highest number of delays. Delays increased gradually up to the 2020 cohort in all three groups, rose sharply with the 2021 cohort, and declined again across the subsequent cohorts without returning to the pre-pandemic level. The relative position of the three groups did not change visibly at any point.

Table 2 shows relative inequalities in developmental delays and their course across cohorts. At the child-weighted mean cohort of the pre-pandemic period, children with moderate (0.711) and high parental education (0.498) exhibited significantly fewer delays than those with low parental education. During the pandemic both ratios increased to 0.786 and 0.582, corresponding to a change of 1.105 and 1.169 and thus to a significant reduction in relative inequality (F(2, 6,863.3) = 5.85, *p* = .003, Table S5). This reduction reflected a significantly stronger increase in developmental delays among children with moderate (1.294) and high parental education (1.369) than among children with low parental education (1.171). Before the pandemic, delays increased in parallel by about three to four% per cohort in all educational groups. This trajectory was interrupted with the 2021 cohort (F(6, 22,905.3) = 8.45, *p* < .001, Table S5), when delays shifted upward before declining again in all educational groups. Since trends ran in parallel both before and during the pandemic (F(2, 3,307.5) = 0.04, *p* = .959, and F(2, 8,720.1) = 2.31, *p* = .100, Table S5), the change in relative inequality was located at the shift in 2021 rather than in diverging trajectories.

**Table 2:** Relative inequalities in developmental delays by parental education and their course across school entry cohorts before and during the pandemic (School entry survey 2015 to 2024, Aachen region, Germany)

	Between educational groups		Within educational groups		
	Moderate vs. low	High vs. low	Low	Moderate	High
	RR (95% CI)	RR (95% CI)	RR (95% CI)	RR (95% CI)	RR (95% CI)
Educational inequalities					
Pre-pandemic period	0.711*** (0.671 – 0.755)	0.498*** (0.460 – 0.538)	–	–	–
Pandemic period	0.786*** (0.742 – 0.832)	0.582*** (0.542 – 0.624)	–	–	–
Change	1.105*	1.169**	1.171***	1.294***	1.369***
	(1.021 – 1.195)	(1.056 – 1.294)	(1.113 – 1.232)	(1.218 – 1.375)	(1.251 – 1.499)
Course over time					
Pre-pandemic trend	1.004 (0.971 – 1.039)	1.005 (0.960 – 1.052)	1.033** (1.011 – 1.055)	1.037** (1.011 – 1.064)	1.038 (0.998 – 1.079)
Pandemic-period trend	0.955 (0.908 – 1.004)	1.020 (0.956 – 1.088)	0.956** (0.925 – 0.989)	0.913*** (0.876 – 0.952)	0.975 (0.918 – 1.035)
Change in trend	0.951 (0.895 – 1.010)	1.015 (0.938 – 1.097)	0.926*** (0.890 – 0.963)	0.880*** (0.838 – 0.924)	0.939 (0.875 – 1.008)
Level shift in 2021	1.233* (1.037 – 1.467)	1.098 (0.881 – 1.368)	1.218*** (1.090 – 1.361)	1.502*** (1.299 – 1.736)	1.337** (1.090 – 1.640)

*CI  * Confidence Interval, *RR  * Rate ratios for the expected number of developmental delays, with 95% confidence intervals from Poisson regression with robust standard errors, estimated across 30 imputed datasets and weighted by stabilized inverse probability weights (*n* = 15,299), adjusted for sex, age at examination, language spoken in the first four years of life, nationality, family structure, kindergarten attendance and late examination in 2021. The pre-pandemic period comprises school entry cohorts 2015–2020 and the pandemic period cohorts 2021–2024. For educational inequalities, both periods are represented by their child-weighted mean cohort, 2017.6 for the pre-pandemic and 2022.6 for the pandemic period. Between-group values are ratios of the corresponding within-group estimates. * *p* < .05, ** *p* < .01, *** *p* < .001. Asterisks indicate significance of individual comparisons and are not corrected for multiple testing. The corresponding joint Wald tests are reported in Table S5. Individual comparisons are therefore descriptive. Low parental education served as reference

Table 3 shows absolute inequalities in developmental delays and their course across cohorts. At the child-weighted mean cohort of the pre-pandemic period, children with low parental education were expected to show 1.766 delays, compared with 1.257 for moderate and 0.879 for high parental education, corresponding to significant differences of -0.510 and -0.887 delays. During the pandemic, developmental delays rose to 2.068 for low, 1.626 for moderate, and 1.204 for high parental education, amounting to a significant increase of 0.302 delays for low, 0.369 for moderate, and 0.324 for high parental education. Since these increases were of comparable size, absolute inequality remained largely unchanged at -0.442 and -0.865, with a nonsignificant change of 0.067 and 0.023 (F(4, 9,763.7) = 1.79, *p* = .128, Table S5). Before the pandemic, delays increased significantly by 0.057 per cohort for low and by 0.046 for moderate parental education, whereas the increase of 0.033 for high parental education was nonsignificant. The 2021 shift was significant in all three groups, amounting to 0.416 delays for low, 0.689 for moderate, and 0.324 for high parental education. Over the subsequent cohorts, delays declined significantly by 0.093 per cohort for low and by 0.148 for moderate parental education, while the decline of 0.031 for high parental education was nonsignificant. None of these estimates differed significantly between educational groups, indicating a similar absolute course of delays across levels of parental education.

**Table 3:** Absolute inequalities in developmental delays by parental education and their course across school entry cohorts before and during the pandemic (School entry survey 2015 to 2024, Aachen region, Germany)

	Between educational groups		Within educational groups		
	Moderate vs. low	High vs. low	Low	Moderate	High
	MD (95% CI)	MD (95% CI)	M (95% CI)	M (95% CI)	M (95% CI)
Educational inequalities					
Pre-pandemic period	-0.510*** (-0.598 – -0.421)	-0.887*** (-0.978 – -0.797)	1.766 (1.699 – 1.833)	1.257 (1.201 – 1.312)	0.879 (0.819 – 0.939)
Pandemic period	-0.442*** (-0.548 – -0.337)	-0.865*** (-0.970 − -0.759)	2.068 (1.989 – 2.147)	1.626 (1.558 – 1.694)	1.204 (1.131 – 1.276)
Change	0.067	0.023	0.302***	0.369***	0.324***
	(-0.063 − 0.198)	(-0.109 − 0.154)	(0.204 – 0.400)	(0.281 – 0.457)	(0.231 – 0.418)
Course over time					
Pre-pandemic trend	-0.011 (-0.061 – 0.039)	-0.024 (-0.076 – 0.028)	0.057** (0.019 – 0.095)	0.046** (0.014 – 0.078)	0.033 (-0.001 − 0.067)
Pandemic-period trend	-0.055 (-0.146 – 0.036)	0.062 (-0.031 – 0.156)	-0.093** (-0.162 − -0.024)	-0.148*** (-0.217 − -0.079)	-0.031 (-0.103 – 0.042)
Change in trend	-0.044 (-0.148 − 0.060)	0.087 (-0.020 − 0.193)	-0.150*** (-0.228 − -0.071)	-0.194*** (-0.270 − -0.118)	-0.063 (-0.142 − 0.016)
Level shift in 2021	0.273 (-0.074 − 0.621)	-0.092 (-0.416 − 0.232)	0.416** (0.171 – 0.662)	0.689*** (0.414 – 0.965)	0.324* (0.078 – 0.571)

*CI  * Confidence Interval, *M  * adjusted mean number of developmental delays, *MD  * difference in adjusted means between educational groups, with negative values indicating fewer delays than in the low educational group. M and MD were estimated as adjusted predictions and contrasts from Poisson regression with robust standard errors, standardized over the covariate distribution of the analytic sample, across 30 imputed datasets and weighted by stabilized inverse probability weights (*n* = 15,299), adjusted for sex, age at examination, language spoken in the first four years of life, nationality, family structure, kindergarten attendance and late examination in 2021. The pre-pandemic period comprises school entry cohorts 2015–2020 and the pandemic period cohorts 2021–2024. For educational inequalities, both periods are represented by their child-weighted mean cohort, 2017.6 for the pre-pandemic and 2022.6 for the pandemic period. Between-group values are differences between the corresponding within-group estimates. * *p* < .05, ** *p* < .01, *** *p* < .001. Asterisks indicate significance of individual comparisons and are not corrected for multiple testing. The corresponding joint Wald tests are reported in Table S5. Individual comparisons are therefore descriptive. Low parental education served as reference

Both main findings held across sensitivity analyses (Tables S6 to S8). The reduction of relative inequality was no longer significant when a nonlinear pandemic trajectory was allowed, and the moderate versus low contrast fell just short of significance when the 2021 cohort was excluded and when the at-risk sample was defined by a constant proportion per cohort. Absolute inequality remained unchanged throughout. Across domains, the reduction of relative inequality was confined to speech and language and visual-motor skills and was no longer significant after correction for multiple testing. No domain showed a significant change on the absolute scale.

## Discussion

This study used repeated cross-sectional data from mandatory SEEs in the Aachen region, Germany, covering children from the cohorts 2015 to 2024 who had been reported by kindergartens or schools as potentially at risk, to examine whether social inequalities in child development changed with the onset of the COVID-19 pandemic. Children of less-educated parents were more likely to show developmental delays throughout the observation period. In the years preceding the pandemic, developmental delays increased to a comparable extent across all educational groups. This trend was interrupted around the onset of the pandemic. Delays rose sharply in all educational groups in the first cohort examined after the pandemic onset and declined again thereafter, significantly only among children with low and moderate parental education. Among children with high parental education, developmental delays remained stable across the observed period apart from the significant shift observed in 2021. Inequalities in developmental delays were stable both before and after the onset of the pandemic, so that the reduction in relative inequality was confined to the shift in 2021 rather than emerging gradually. It reflected a proportionally larger increase among the better-educated groups. On the absolute scale, by contrast, the difference between the educational groups remained stable throughout.

Children of less-educated parents exhibited a substantially higher expected number of developmental delays across both periods, an educational gradient that persisted consistently across all ten cohorts examined. The magnitude of this gradient aligns closely with estimates reported for other German and European populations [20–22, 29]. Several mechanisms plausibly contribute to inequalities in developmental delays [57]. The family investment model attributes disparities in developmental delays to systematic variations in the material and cognitive resources parents allocate to their children [58]. It also encompasses the use of available services, such as participation in preventive pediatric examinations and enrollment in early childhood education, both of which are less frequent among socially disadvantaged families [30, 59]. Moreover, the family stress model emphasizes the detrimental impact of economic hardship on parental well-being and, consequently, on the responsiveness of parent-child interactions [60]. Evidence indicates that these two pathways operate jointly rather than as mutually exclusive alternatives [61, 62]. Additional social processes may drive these patterns, including the residential and institutional segregation of disadvantaged families [63–65]. Moreover, institutionalized discrimination against non-traditional families can publicly discredit disadvantaged parents [8, 66]. Social prejudices may also influence how school physicians assess children, including through perceptions of their social background and conformity with institutional expectations [67, 68]. All of these mechanisms may help explain the gradient rather than its change over time, which is consistent with the absence of any systematic drift in inequality within either the pre-pandemic or the pandemic period.

Superimposed on this stable gradient was a general increase in developmental delays that affected all educational groups to a similar extent and persisted after adjustment for the changing composition of the cohorts. Comparable increases appear in other German school entry populations and in routine health insurance data [69, 70]. Multiple factors may have contributed to this trend. The institutional context in Germany has changed over the past decade, including an increasing emphasis on prevention and early detection of developmental difficulties as well as a marked expansion of early childhood education and care [71, 72]. This shift may have heightened awareness and sensitivity to child development among kindergarten teachers, SEE staff, and examining physicians, potentially increasing the identification of both children at risk and actual developmental delays. However, staffing ratios remained below scientifically recommended levels, which may have contributed to the increase by adversely affecting children’s developmental environments [72]. Finally, screen media exposure in early childhood has become more widespread worldwide and in Germany [73, 74], and prospective studies have linked such exposure to poorer performance on developmental screening, including in the domains of communication and problem solving [75, 76]. These mechanisms could explain a population-wide upward shift in developmental delays while leaving the underlying educational gradient largely unchanged.

Around the onset of the pandemic, this developmental trajectory was disrupted rather than accelerated. Delays increased sharply in the first cohort examined after the onset, a finding consistent with evidence from other countries, where similar changes have been linked to factors such as the closure of early childhood institutions, restrictions on contact with peers, and reduced opportunities for physical activity and structured learning [2–4]. The increase took the form of a discontinuity, as the elevated level during the pandemic period was driven primarily by the abrupt shift observed in the 2021 cohort rather than by a continuation or acceleration of the pre-pandemic trend. The analysis therefore identifies a discontinuity coinciding with the pandemic rather than an effect attributable to it alone, since containment measures, childcare closures, examination procedures, and referral practices changed at the same time. Because the 2021 cohort was examined under a modified selection rule, this discontinuity could in part reflect differences in ascertainment rather than a change in developmental outcomes. The principal findings were nevertheless robust in direction across five alternative definitions of the at-risk sample and when the 2021 cohort was excluded, although the moderate versus low contrast fell just short of significance in two of these analyses. Delays declined again in subsequent cohorts, although this decline was statistically significant only among children with low and moderate parental education, and in none of the groups did delays return to their pre-pandemic level.

The results indicate that relative inequality narrowed significantly between the pre-pandemic and the pandemic period, whereas the absolute difference between educational groups remained unchanged. Sensitivity analyses show that these findings rest largely on delays in speech and language and, less robustly, on visual-motor skills, although neither contrast remained significant after correction for multiple testing. Both depend more directly than the remaining domains on sustained interaction and structured practice, which closures and contact restrictions curtailed, although the rarer domains were also estimated with less precision. However, the narrowing on the relative scale may be a mathematical consequence of differing baseline levels rather than a substantive reduction in disadvantage. As developmental delays were up to twice as frequent among children of less-educated parents before the pandemic, a similar absolute increase amounted to a smaller proportional rise in this group than among children with moderate or high parental education. That relative and absolute measures can move in opposite directions whenever baseline levels differ is well established, and conclusions about widening or narrowing remain uninformative unless the scale is specified [31, 32].

Studies from Uruguay and from the Hanover region in Germany reported greater pandemic-related losses among socioeconomically disadvantaged preschool children [33, 77], which appears to contradict the present findings. Part of the discrepancy may reflect differences in the developmental domains assessed, in the indicator of social position, in the children’s age, in the measurement procedure, and in the pandemic period covered, and part of it the scale on which inequality was expressed. Analyses of other health outcomes during the pandemic show a similar dependence, with the direction and magnitude of change in socioeconomic inequality varying across outcomes [78]. School entry data from Düsseldorf, Germany, by contrast, point in the same direction as the present results, with language problems increasing more strongly in socioeconomically advantaged neighborhoods [29]. While it cannot be ruled out that the pandemic had a smaller impact on children of parents with lower levels of education, the data do not require this assumption, and any such effect would have to be so small that it remained undetected in the absolute comparison. The abrupt increase in developmental delays in the first cohort examined after the pandemic onset was robust across model specifications. In contrast, the magnitude and persistence of the change in relative inequality were less robust, because allowing for a nonlinear trajectory after the 2021 cohort attenuated the estimated change so that it was no longer statistically significant. The results therefore provide stronger evidence for a marked discontinuity around the onset of the pandemic than for a precise estimate of the magnitude or duration of the change in inequality.

A primary strength is the population-based series of ten consecutive school entry cohorts, four of them following the onset of the pandemic, which allows an abrupt shift in level to be distinguished from a change in trend and the parallelism of pre-pandemic trajectories to be tested rather than assumed. Participation is mandatory, and development was assessed by trained public health professionals using standardized instruments rather than by parental report. Inequality was quantified on both the relative and the absolute scale, and multiple imputation, inverse probability weighting, correction for multiple testing, and various sensitivity analyses were applied.

However, several limitations must be acknowledged. First, the analysis was limited to children identified as being at greater risk for developmental delays prior to the SEE, and results therefore cannot be generalized to the full school entry cohort. The estimates describe how delays and their social gradient developed among children brought to attention before the examination, not how frequent delays were in the cohort as a whole. Since this identification was related to the outcome analyzed, selection bias cannot be ruled out [49]. The weights balance measured characteristics across cohorts but cannot correct for changes in the thresholds that kindergarten and school staff applied when reporting children. Although the findings remained stable across sensitivity analyses, a residual effect is particularly plausible in the 2021 cohort, where altered examination strategies, pandemic-related closures, and reduced attendance constrained professionals’ observation opportunities. Second, the identification of at-risk children relied on observations by kindergarten and school staff rather than systematic screening, which may introduce biases driven by institutional expectations. Third, since the study used school entry cohorts over time without a separate control group, the effects of the pandemic cannot be separated from other concurrent changes. Therefore, the observed shift in 2021 reflects everything that changed during that period, not only the implemented mitigation measures. Fourth, although standardized instruments were used, the recording of a delay involves professional judgment, and shifts in diagnostic attention over the observation period cannot be excluded. Fifth, the segmented model assumes a linear trend within each period. When a nonlinear pandemic trajectory was permitted, the reduction in relative inequality was attenuated and no longer statistically significant, indicating that this finding is sensitive to the functional form assumed for the post-pandemic period (see supplementary Table S6). Finally, the outcome counts delayed domains without weighting them, parental education was the only available indicator of social position, and socioemotional development and household income were not recorded. The data also derive from a single region, which limits transferability to areas with a different social composition.

## Conclusion

This ten-year analysis of SEEs shows that inequalities in child development by parental education remained remarkably persistent throughout the COVID-19 pandemic. Children of less-educated parents carried a consistently higher burden of developmental delays. The gradient narrowed modestly on the relative scale, but only because delays rose proportionally more among the better-educated groups, while the absolute gap between the groups remained unchanged. The dominant feature of the period was an isolated and abrupt disruption in the first cohort examined after the pandemic onset, followed by a partial recovery that left overall levels elevated compared with the pre-pandemic years. These findings highlight that while broad societal shocks and changing institutional practices can trigger sudden population-wide shifts in identified developmental risks, addressing deep-rooted socioeconomic disparities requires sustained structural interventions rather than temporary crisis management.

## Supplementary Information

Supplementary Material 1.

## Data Availability

The data are available upon request at the Health Authority of the City and Area of Aachen, Aachen, Germany.
