# Supplementary material for: Has the COVID-19 pandemic altered social inequalities in child development? A time trend analysis of developmental delays in preschool children by parental education
Source: Arch Public Health. 2026 Oct 15;84:223. doi: 10.1186/s13690-026-02111-x (PMC13670839; doi:10.1186/s13690-026-02111-x)
Supplement: Supplementary file 1 [file 13690_2026_2111_MOESM1_ESM.docx]

| **Table S1:** Composition of the analytic sample with and without multiple imputation and inverse probability weighting, and in the full school entry cohort (School entry survey 2015 to 2024, Aachen region, Germany) | | | | | |
| --- | --- | --- | --- | --- | --- |
|  | **Analytical Sample (at risk)** | | | | **Benchmark** |
|  | **Raw** | **Weighted^a^** | **Imputed** | **Both** | **Full cohort** |
| Parental education |  |  |  |  |  |
| Low | 33.6 (30.8 – 39.2) | 19.6 (17.6 – 22.2) | 34.4 (31.7 – 40.8) | 21.1 (19.1 – 23.1) | 21.2 (19.3 – 23.0) |
| Moderate | 38.5 (34.2 – 44.2) | 39.2 (37.9 – 41.5) | 38.1 (33.3 – 43.7) | 38.5 (36.8 – 41.3) | 38.3 (36.0 – 40.6) |
| High | 27.9 (22.0 – 33.5) | 41.2 (36.2 – 44.1) | 27.5 (21.7 – 32.9) | 40.4 (36.3 – 43.5) | 40.5 (36.6 – 43.3) |
| Missing | 9.6 (6.1 – 14.8) | — | — | — | — |
| Sex |  |  |  |  |  |
| Male | 58.6 (54.8 – 63.1) | 51.5 (47.7 – 59.2) | 58.6 (54.8 – 63.1) | 51.4 (47.5 – 57.8) | 51.9 (50.6 – 53.5) |
| Female | 41.4 (37.0 – 45.2) | 48.5 (40.8 – 52.3) | 41.4 (37.0 – 45.2) | 48.6 (42.2 – 52.5) | 48.1 (46.5 – 49.4) |
| Nationality |  |  |  |  |  |
| German | 83.3 (78.5 – 90.6) | 88.7 (84.0 – 93.4) | 82.4 (77.2 – 90.1) | 87.2 (82.7 – 92.7) | 87.6 (84.6 – 90.5) |
| Other | 16.7 (9.4 – 21.5) | 11.3 (6.6 – 16.0) | 17.7 (9.9 – 22.8) | 12.8 (7.3 – 17.3) | 12.4 (9.6 – 15.4) |
| Missing | 8.8 (4.0 – 25.2) | — | — | — | — |
| Language in the first 4 life years |  |  |  |  |  |
| German | 52.5 (46.6 – 61.4) | 59.1 (53.9 – 62.5) | 52.4 (46.6 – 61.0) | 56.2 (50.5 – 62.7) | 61.8 (45.3 – 65.8)^b^ |
| Other | 47.5 (38.6 – 53.4) | 40.9 (37.5 – 46.1) | 47.6 (39.0 – 53.4) | 43.8 (37.3 – 49.5) | 38.2 (34.2 – 54.7)^b^ |
| Missing | 1.6 (0.0 – 6.4) | 0.6 (0.0 – 2.4) | — | — | — |
| Family structure |  |  |  |  |  |
| Natural parents | 75.5 (71.4 – 77.8) | 80.7 (77.7 – 82.4) | 75.4 (71.3 – 77.9) | 80.6 (77.8 – 82.1) | 80.5 (78.6 – 82.4) |
| Single parent | 17.6 (14.7 – 20.5) | 14.0 (12.1 – 16.0) | 17.7 (14.9 – 20.6) | 14.0 (12.1 – 15.8) | 14.1 (12.9 – 15.1) |
| Natural parent with partner | 4.5 (3.2 – 5.7) | 4.0 (3.1 – 5.0) | 4.5 (3.2 – 5.7) | 4.0 (3.0 – 5.1) | 4.0 (2.8 – 4.7) |
| Children's home/Caregivers | 1.8 (0.9 – 3.0) | 1.0 (0.6 – 1.8) | 1.8 (0.9 – 3.1) | 1.1 (0.6 – 1.8) | 1.1 (0.7 – 1.7) |
| Others | 0.5 (0.3 – 0.9) | 0.3 (0.2 – 0.4) | 0.5 (0.3 – 0.9) | 0.3 (0.2 – 0.6) | 0.3 (0.2 – 0.5) |
| Missing | 7.0 (5.2 – 10.0) | — | — | — | — |
| Kindergarten attendance |  |  |  |  |  |
| Yes | 94.4 (91.0 – 97.4) | 95.9 (94.3 – 97.2) | 94.4 (91.0 – 97.4) | 94.3 (90.9 – 96.9) | 94.4 (90.4 – 97.4) |
| No | 5.6 (2.6 – 9.0) | 4.1 (2.8 – 5.7) | 5.6 (2.6 – 9.0) | 5.7 (3.1 – 9.2) | 5.6 (2.6 – 9.6) |
| Developmental delays (0 – 6), mean | 1.44 (1.19 – 1.66) | 1.25 (1.05 – 1.48) | 1.56 (1.30 – 1.79) | 1.36 (1.12 – 1.62) | 0.76 (0.63 – 0.89)^c^ |
| Age at examination (months), mean | 67.99 (67.35 – 69.66) | 67.82 (67.16 – 69.09) | 67.99 (67.35 – 69.66) | 67.91 (67.26 – 69.25) | 70.88 (70.45 – 71.44)^c^ |
|  | | | | | |

*Notes:* Values are percentages within the analytic sample (n = 15,299), with the range across the ten school entry cohorts in parentheses. **Raw** = observed data, unweighted. **Weighted** = observed data with stabilized inverse probability weights. **Imputed** = 30 imputed datasets, unweighted. **Both** = imputed datasets with weights used in all analyses reported in the main text. **Full cohort** = corresponding distribution among all children of the ten cohorts (N = 48,121), pooled across imputations. Categories sum to 100 excluding missing values, which are reported separately as a share of all cases. ^a^Weights could not be computed for 2,703 children with incomplete covariates, so the weighted column rests on 12,596 cases. Characteristics entering the weighting model therefore show no missing values in that column. ^b^In the 2021 cohort, language was recorded only for the children who were examined. For the remainder it is imputed from a stratum without observed values, which explains the low proportion of German-speaking children in that year (45.3% versus 61 – 66% in all other cohorts). ^c^The full cohort also includes children never reported as at risk, who have fewer delays and are examined later in the school year and therefore at an older age.

**Table S2:** Diagnostic test and qualitative assessment of child development in the school entrance examination of the Aachen region, Germany.

| **Area** | **Task** | **Results of the Task^a^** | **Qualitative assessment** |
| --- | --- | --- | --- |
| **Physical coordination** | - Jumping sideways back and forth across a centre line within a given area in a period of 10 seconds. | Conspicuous: 0 - 6 jumps  Moderate: 7 - 8 jumps  Inconspicuous: from 9 jumps | - The child has a rigid and tense or limp posture. - The child visibly finds it difficult to keep his balance. - The child shows large balancing movements, does not land safely. - The child does not cushion the jump in the knees, movements are jerky. - The child needs a lot of strength and effort for the task. - The child has difficulty keeping to the boundary (many oversteps). |
| **Selective attention** | - Cross-out task of sad faces with time limit | Conspicuous: 0 – 10 points  Moderate: 11 – 13 points  Inconspicuous: 14 – 29 points | - Does a child stay on task? - How does it deal with instructions? - Does behaviour change during the course of the examination? |
| **Visual-motor skills** | - completing missing geometric shapes of a pre-drawn picture - free drawing of two geometric figures from a template. | Conspicuous: 0 – 4 points  Moderate: 5 – 6 points  Inconspicuous: 7 – 12 points | - Does a child stay on task? - How does it deal with instructions? - Does the behaviour change in the course of the examination? - Does the child make involuntary compensatory movements? - Is the dosage of force appropriate? |
| **Visual perception and reasoning** | - finding a suitable wagon for a train or identifying a wagon that does not fit on a represented train. | Conspicuous: 0 – 8 points  Moderate: 9 – 10 points  Inconspicuous: 11 – 15 points | - Does a child stay on task? - Which items are selected? - How does it deal with instructions? - Does the behaviour change in the course of the examination? |
| **Prior knowledge of numbers and quantities** | *Counting test*:   - count out 20 balls on a card | Conspicuous: 0 – 12 points  Moderate: 13 – 16 points  Inconspicuous: 17 – 20 points | - Does a child stay on task? - How does it deal with instructions? - Does the behaviour change in the course of the examination? |
|  | *Quantitative knowledge test:*   - simultaneous detection of a number of balls with time limit - distinguish quantities of balls | Conspicuous: 0 – 10 points  Moderate: 11 – 13 points  Inconspicuous: 14 – 16 points |  |
| **Speech and language** | *Plural formation test:*   - naming the plural form of terms | Conspicuous: 0 - 4 points  Moderate: 5 points  Inconspicuous: 6 to 8 points | - Does the child show disorders such as stammering, poluttering and other speech disorders or nasal twang |
|  | *Preposition test:*   - describing an image and correctly locating objectives in relation to each other in the image | Conspicuous: 0 – 3 points  Moderate: 4 - 5 points  Inconspicuous: 6 – 7 points |  |
|  | *Pseudoword test:*   - repeating of given pseudo words | Conspicuous: 0 – 3 points  Moderate: 4 points  Inconspicuous: 5 – 6 points |  |
|  | - recording of articulation errors |  |  |
| ^a^The cut-off values were based on a norm sample in which the test-results identified as conspicuous can be assigned to the lowest 10th percentile in the norm sample. A moderate test result lies within the 10th and 25th percentile in the norm sample, while results above the 25th percentile in the norm sample are described as non-conspicuous. | | | |

**Table S3:** Scores for the combination of the highest educational and vocational degree to determine parental education.

|  |  | **Educational degree** | | | | | |
| --- | --- | --- | --- | --- | --- | --- | --- |
|  |  | No educational degree | Secondary School (*Hauptschulabschluss*) | Intermediate school (*Realschulabschluss*) | Vocational diploma (*Fachholschulreife)* | High school diploma (*Abitur*) | Other degree |
| **Vocational degree** | No vocational degree | 1 | 2 | 3 | 5 | 6 | 1 |
|  | Vocational training | 3 | 3 | 4 | 5 | 6 | 3 |
|  | Vocational-school training | 3 | 3 | 4 | 5 | 6 | 3 |
|  | Technical school (*Fachschule*) | - | 4 | 5 | 6 | 6 | 4 |
|  | University of Applied Sciences (*Fachhochschule*) | - | - | 7 | 7 | 7 | 7 |
|  | University | - | - | 8 | 8 | 8 | 8 |
|  | Other degree | 1 | 3 | 4 | 5 | 7 | 3 |

| **Table S4:** Expected number of developmental delays by school entry cohort and parental education (School entry survey 2015 to 2024, Aachen region, Germany) | | | | | | |
| --- | --- | --- | --- | --- | --- | --- |
|  | **Panel A: unadjusted** | | | **Panel B: adjusted** | | |
| **School entry cohort** | **Low** | **Moderate** | **High** | **Low** | **Moderate** | **High** |
| 2015 | 1.73 (1.55 – 1.91) | 1.10 (0.97 – 1.24) | 0.75 (0.62 – 0.88) | 1.68 (1.51 – 1.86) | 1.12 (0.99 – 1.26) | 0.77 (0.64 – 0.90) |
| 2016 | 1.67 (1.51 – 1.83) | 1.14 (1.01 – 1.27) | 0.90 (0.72 – 1.08) | 1.58 (1.43 – 1.73) | 1.15 (1.02 – 1.28) | 0.90 (0.72 – 1.08) |
| 2017 | 1.83 (1.66 – 2.00) | 1.29 (1.15 – 1.43) | 0.88 (0.72 – 1.03) | 1.78 (1.62 – 1.95) | 1.31 (1.17 – 1.44) | 0.90 (0.74 – 1.06) |
| 2018 | 1.78 (1.63 – 1.94) | 1.26 (1.13 – 1.39) | 0.68 (0.56 – 0.80) | 1.69 (1.55 – 1.84) | 1.26 (1.13 – 1.38) | 0.69 (0.56 – 0.81)* |
| 2019 | 1.91 (1.75 – 2.06) | 1.27 (1.14 – 1.41) | 1.02 (0.87 – 1.18)* | 1.82 (1.67 – 1.96) | 1.27 (1.14 – 1.40) | 1.04 (0.88 – 1.19)* |
| 2020 | 2.18 (2.02 – 2.35)* | 1.41 (1.26 – 1.55) | 0.93 (0.80 – 1.06) | 1.96 (1.82 – 2.11) | 1.38 (1.24 – 1.51) | 0.95 (0.83 – 1.08) |
| 2021 | 2.13 (1.98 – 2.28) | 1.79 (1.62 – 1.95)* | 1.21 (1.05 – 1.36)* | 2.65 (2.44 – 2.86)* | 2.27 (2.04 – 2.51)* | 1.54 (1.33 – 1.76)* |
| 2022 | 2.16 (2.01 – 2.31) | 1.57 (1.43 – 1.72) | 1.15 (1.00 – 1.30) | 1.96 (1.83 – 2.10)* | 1.54 (1.40 – 1.67)* | 1.10 (0.96 – 1.24)* |
| 2023 | 2.06 (1.91 – 2.21) | 1.50 (1.38 – 1.63) | 1.15 (1.02 – 1.28) | 1.88 (1.74 – 2.01) | 1.45 (1.34 – 1.57) | 1.11 (0.99 – 1.23) |
| 2024 | 2.30 (2.15 – 2.44)* | 1.61 (1.49 – 1.73) | 1.27 (1.13 – 1.41) | 2.08 (1.94 – 2.21)* | 1.54 (1.43 – 1.65) | 1.23 (1.10 – 1.37) |
| *Notes:* Values are model - based predictions of the expected number of developmental delays (range 0 – 6) with 95% confidence intervals, weighted by stabilized inverse probability weights and pooled across 30 imputed datasets. Panel A is unadjusted. Panel B is adjusted for sex, age at examination, language spoken in the first four years of life, nationality, family structure, kindergarten attendance and late examination in the 2021 cohort, and standardized over the covariate distribution of the analytic sample. *change from the preceding cohort at p ≤ .05. Comparisons are exploratory and not corrected for multiple testing, and correspond to the line style in Figure 1. | | | | | | |

| **Table S5:** Joint Wald tests of the time structure and of the educational gradient in developmental delays (School entry survey 2015 to 2024, Aachen region, Germany) | | | | |
| --- | --- | --- | --- | --- |
| **Test** | **Null hypothesis** | **Model terms tested** | **F (df1, df2)** | ***p*** |
| T1 | Educational inequality in developmental delays was constant across pre-pandemic cohorts | Education × cohort trend | 0.04 (2, 3,307.5) | 0.959 |
| T2 | The pre-pandemic trend continued unchanged from the 2021 cohort onwards, in all educational groups | Level shift and change in trend, each as a main effect and in interaction with education | 8.45 (6, 22,905.3) | < 0.001 |
| T3 | The level shift in the 2021 cohort and the change in trend thereafter were identical across educational groups | Education × level shift; education × change in trend | 1.79 (4, 9,763.7) | 0.128 |
| T3a | The level shift in the 2021 cohort was identical across educational groups | Education × level shift | 2.82 (2, 3,875.6) | 0.060 |
| T3b | The change in trend was identical across educational groups | Education × change in trend | 1.75 (2, 7,774.2) | 0.174 |
| T4 | Educational inequality in developmental delays was constant across pandemic-period cohorts | Education × pandemic-period trend | 2.31 (2, 8,720.1) | 0.100 |
| *Notes:* n = 15,299 children in the at-risk sample. All tests are multiple-imputation Wald tests (M = 30) following the Poisson regression reported in Table 2, in which the number of developmental delays is regressed on parental education interacted with a linear cohort trend, a level shift for the 2021 cohort and a change in trend thereafter, adjusted for sex, age at examination, language spoken in the first four years of life, nationality, family structure, kindergarten attendance and late examination in 2021, and weighted by stabilized inverse probability weights. Denominator degrees of freedom are adjusted for the fraction of missing information and therefore differ across tests. T3a and T3b decompose T3 into the level component and the slope component. Parental education was coded as low (reference), moderate and high. | | | | |

| **Table S6:** Sensitivity analyses for the change in educational inequality in developmental delays between the pre-pandemic and the pandemic period (School entry survey 2015 to 2024, Aachen region, Germany) | | | | | |
| --- | --- | --- | --- | --- | --- |
| **Analysis** | **n** | **RRR moderate vs. low** | **RRR high vs. low** | **Change in MD moderate vs. low** | **Change in MD high vs. low** |
| Main analysis | 15,299 | 1.105* (1.021 – 1.195) | 1.169** (1.056 – 1.294) | 0.067 (-0.063 – 0.198) | 0.023 (-0.109 – 0.154) |
| Complete cases instead of multiple imputation | 10,847 | 1.111* (1.015 – 1.216) | 1.221** (1.082 – 1.376) | 0.078 (-0.063 – 0.218) | 0.063 (-0.080 – 0.206) |
| Without inverse probability weighting | 15,299 | 1.085* (1.006 – 1.170) | 1.125* (1.023 – 1.239) | 0.040 (-0.091 – 0.172) | −0.020 (-0.153 – 0.113) |
| Excluding the 2021 cohort | 13,878 | 1.091 (0.996 – 1.195) | 1.159* (1.031 – 1.302) | 0.075 (-0.075 – 0.225) | 0.064 (-0.088 – 0.216) |
| At risk by 28 February | 20,107 | 1.118** (1.037 – 1.205) | 1.222*** (1.112 – 1.343) | 0.065 (-0.044 – 0.174) | 0.044 (-0.063 – 0.151) |
| At risk by 31 December | 10,452 | 1.129** (1.035 – 1.232) | 1.199** (1.066 – 1.348) | 0.154 (-0.014 – 0.321) | 0.145 (-0.034 – 0.325) |
| At risk by a constant proportion per cohort | 15,295 | 1.080 (0.997 – 1.171) | 1.142* (1.030 – 1.265) | 0.017 (-0.116 – 0.150) | −0.029 (-0.161 – 0.104) |
| At risk by 28 February in the 2021 cohort only | 14,833 | 1.093* (1.008 – 1.185) | 1.130* (1.018 – 1.253) | 0.047 (-0.090 – 0.183) | −0.026 (-0.161 – 0.109) |
| Allowing a non-linear pandemic trajectory | 15,299 | 1.050 (0.928 – 1.188) | 1.155 (0.982 – 1.358) | 0.029 (-0.162 – 0.220) | 0.068 (-0.128 – 0.264) |
| *Notes*: CI = confidence interval. RRR = ratio of rate ratios, expressing the change in relative inequality between the pre-pandemic and the pandemic period. Change in MD = change in the difference in adjusted mean number of developmental delays between educational groups. All estimates are from Poisson regression with robust standard errors, adjusted for the covariates listed in Table 2. Values in parentheses are 95% confidence intervals. * p < .05, ** p < .01, *** p < .001. Low parental education served as reference. | | | | | |

| **Table S7**: Change in relative educational inequality between the pre-pandemic and the pandemic period, by domain of child development (School entry survey 2015 to 2024, Aachen region, Germany) | | | | | | | |
| --- | --- | --- | --- | --- | --- | --- | --- |
| **Domain** | **Comparison** | **Pre-pandemic PR** | **Pandemic PR** | **Change** | **p** | **p Holm** | **p BH** |
| Physical coordination | Moderate vs. low | 0.817** (0.724–0.923) | 0.870* (0.770–0.983) | 1.064 (0.901–1.257) | 0.465 | 1.000 | 0.558 |
|  | High vs. low | 0.666*** (0.574–0.773) | 0.750*** (0.652–0.862) | 1.125 (0.923–1.371) | 0.243 | 1.000 | 0.364 |
| Selective attention | Moderate vs. low | 0.660*** (0.585–0.746) | 0.721*** (0.638–0.816) | 1.092 (0.925–1.291) | 0.299 | 1.000 | 0.398 |
|  | High vs. low | 0.448*** (0.382–0.526) | 0.474*** (0.403–0.557) | 1.058 (0.850–1.317) | 0.614 | 1.000 | 0.670 |
| Visual-motor skills | Moderate vs. low | 0.697*** (0.648–0.751) | 0.794*** (0.738–0.853) | 1.138 (1.031–1.256) | 0.010 | 0.101 | 0.041 |
|  | High vs. low | 0.442*** (0.399–0.491) | 0.517*** (0.470–0.568) | 1.168 (1.020–1.338) | 0.024 | 0.220 | 0.062 |
| Visual perception | Moderate vs. low | 0.590*** (0.515–0.674) | 0.614*** (0.527–0.716) | 1.042 (0.857–1.267) | 0.681 | 1.000 | 0.681 |
|  | High vs. low | 0.379*** (0.314–0.456) | 0.453*** (0.377–0.545) | 1.197 (0.930–1.541) | 0.163 | 1.000 | 0.318 |
| Numbers and quantities | Moderate vs. low | 0.546*** (0.470–0.634) | 0.619*** (0.543–0.705) | 1.134 (0.942–1.365) | 0.185 | 1.000 | 0.318 |
|  | High vs. low | 0.355*** (0.292–0.432) | 0.465*** (0.399–0.543) | 1.311 (1.033–1.664) | 0.026 | 0.220 | 0.062 |
| Speech and language | Moderate vs. low | 0.791*** (0.753–0.830) | 0.881*** (0.842–0.921) | 1.114 (1.045–1.187) | 0.001 | 0.010 | 0.006 |
|  | High vs. low | 0.577*** (0.539–0.618) | 0.688*** (0.650–0.729) | 1.193 (1.093–1.302) | < 0.001 | 0.001 | 0.001 |
| Notes: CI = confidence interval. PR = prevalence ratio for a delay in the respective domain, with low parental education as reference. Change = ratio of the prevalence ratios between the pre-pandemic and the pandemic period. All estimates are from Poisson regression with robust standard errors, estimated across 30 imputed datasets and weighted by stabilized inverse probability weights (n = 15,299), adjusted for the covariates listed in Table 2. Values in parentheses are 95% confidence intervals. Asterisks refer to the prevalence ratios and indicate whether inequality differed from unity within the respective period, uncorrected for multiple testing: * p < .05, ** p < .01, *** p < .001. The three p-value columns refer to the change: p is uncorrected, whereas p Holm and p BH are corrected across all twelve comparisons using the Holm and the Benjamini-Hochberg procedure. | | | | | | | |

| **Table S8**: Change in absolute educational inequality between the pre-pandemic and the pandemic period, by domain of child development (School entry survey 2015 to 2024, Aachen region, Germany) | | | | | | | |
| --- | --- | --- | --- | --- | --- | --- | --- |
| **Domain** | **Comparison** | **Pre-pandemic PR** | **Pandemic PR** | **Change** | **p** | **p Holm** | **p BH** |
| Physical coordination | Moderate vs. low | -0.032** (-0.052 – -0.013) | -0.029* (-0.054 – -0.003) | 0.004 (-0.027 – 0.035) | 0.808 | 1.000 | 0.889 |
|  | High vs. low | -0.059*** (-0.080 – -0.038) | -0.055*** (-0.082 – -0.029) | 0.004 (-0.028 – 0.037) | 0.796 | 1.000 | 0.889 |
| Selective attention | Moderate vs. low | -0.067*** (-0.087 – -0.048) | -0.067*** (-0.093 – -0.041) | 0.000 (-0.031 – 0.031) | 0.996 | 1.000 | 0.996 |
|  | High vs. low | -0.110*** (-0.130 – -0.089) | -0.127*** (-0.153 – -0.101) | -0.018 (-0.048 – 0.013) | 0.265 | 1.000 | 0.558 |
| Visual-motor skills | Moderate vs. low | -0.122*** (-0.147 – -0.097) | -0.099*** (-0.130 – -0.068) | 0.024 (-0.014 – 0.061) | 0.222 | 1.000 | 0.558 |
|  | High vs. low | -0.225*** (-0.250 – -0.200) | -0.231*** (-0.262 – -0.200) | -0.006 (-0.044 – 0.032) | 0.756 | 1.000 | 0.889 |
| Visual perception | Moderate vs. low | -0.076*** (-0.095 – -0.057) | -0.073*** (-0.096 – -0.050) | 0.003 (-0.025 – 0.032) | 0.815 | 1.000 | 0.889 |
|  | High vs. low | -0.115*** (-0.135 – -0.096) | -0.103*** (-0.125 – -0.081) | 0.012 (-0.015 – 0.040) | 0.388 | 1.000 | 0.664 |
| Numbers and quantities | Moderate vs. low | -0.081*** (-0.100 – -0.061) | -0.097*** (-0.123 – -0.071) | -0.017 (-0.047 – 0.014) | 0.279 | 1.000 | 0.558 |
|  | High vs. low | -0.115*** (-0.133 – -0.096) | -0.136*** (-0.162 – -0.111) | -0.022 (-0.051 – 0.007) | 0.142 | 1.000 | 0.558 |
| Speech and language | Moderate vs. low | -0.130*** (-0.157 – -0.103) | -0.082*** (-0.110 – -0.053) | 0.048 (0.011 – 0.086) | 0.011 | 0.135 | 0.106 |
|  | High vs. low | -0.263*** (-0.293 – -0.234) | -0.213*** (-0.244 – -0.183) | 0.050 (0.009 – 0.091) | 0.018 | 0.194 | 0.106 |
| Notes: CI = confidence interval. PR = prevalence ratio for a delay in the respective domain, with low parental education as reference. Change = ratio of the prevalence ratios between the pre-pandemic and the pandemic period. All estimates are from Poisson regression with robust standard errors, estimated across 30 imputed datasets and weighted by stabilized inverse probability weights (n = 15,299), adjusted for the covariates listed in Table 2. Values in parentheses are 95% confidence intervals. Asterisks refer to the prevalence ratios and indicate whether inequality differed from unity within the respective period, uncorrected for multiple testing: * p < .05, ** p < .01, *** p < .001. The three p-value columns refer to the change: p is uncorrected, whereas p Holm and p BH are corrected across all twelve comparisons using the Holm and the Benjamini-Hochberg procedure. | | | | | | | |
